# Editorial: Reproductive biotechnologies and challenges in their application

**DOI:** 10.3389/fvets.2025.1558641

**Published:** 2025-02-04

**Authors:** Stefan G. Ciornei, Graça Lopes, Mihai Cenariu

**Affiliations:** ^1^Faculty of Veterinary Medicine, Ion Ionescu de la Brad University of Life Sciences Iasi, Iasi, Romania; ^2^School of Medicine and Biomedical Sciences (ICBAS), University of Porto, Porto, Portugal; ^3^Faculty of Veterinary Medicine, University of Agricultural Sciences and Veterinary Medicine of Cluj-Napoca, Cluj-Napoca, Romania

**Keywords:** reproductive biotechnologies, embryo, semen cryopreservation, reproductive endocrinology, postpartum management

*Reproductive biotechnologies and challenges in their application*, a Research Topic hosted by Frontiers in Veterinary Science (Animal Reproduction—Theriogenology) was launched in February 2024. The aim was to explore the advancements made by researchers in the field of reproductive biotechnologies, assess their potential for improving reproductive health and efficiency, and address the challenges associated with their ethical, technical, and practical applications. This research is fundamental for the progress of animal reproductive health and productivity, by addressing infertility challenges, and ensuring the ethical and sustainable application of state-of-the-art biotechnological solutions in this field. Thus, 17 original research papers, as well as a brief research report were published.

Several papers focused on embryology across various species, including bovines, equines, swine, and monkeys.

Jung et al. evaluated the efficiency of slow freezing bovine blastocysts with sucrose added prior to freezing, to overcome ice crystal formation caused by insufficient dehydration (1). The study revealed that treating bovine embryos with 0.25 M sucrose before slow freezing improved their post freeze-thawing viability.

Antral follicle count (AFC) is a crucial factor in bovine embryo production and donor selection (2). Gawai et al. assessed the effect of AFC on follicular and luteal development during the estrous cycle and superovulatory period, as well as on superovulatory response and *in vivo* embryo quality in Sahiwal cows. Their research confirmed that an AFC exceeding 30 is a reliable phenotypic marker for predicting the reproductive potential of Sahiwal donors, making it significant for commercial *in vivo* embryo production programs.

Lipids play a key role in embryo-maternal signaling and early embryo development (3). Lawson et al. conducted lipidomic profiling of early equine embryos *in vitro*, revealing that triglycerides were consistently released into the culture environment, while diglycerides were depleted. This highlights the importance of a well-defined embryo culture medium.

Myo-inositol is known for its protective role against oxidative stress via the NRF2/KEAP1 signaling pathway (4). Jawad et al. investigated its effect on porcine embryonic development after parthenogenetic activation. Findings demonstrated that 20 mM Myo-inositol supplementation of culture media enhanced blastocyst development and improved mitochondrial function by regulating apoptosis, reducing oxidative stress, and activating the NRF2 pathway.

Nonhuman primates are critical for generating gene-edited models for human disease research (5). Lee et al. investigated the efficacy of superovulated and uterine-embryo synchronized recipients of embryo transfer in cynomolgus monkeys. Outcomes confirmed that superovulated recipients were as effective as synchronized ones, facilitating efficient gene-edited model generation.

Various papers focused on spermology, providing insights into semen cryopreservation in several species.

Avdatek et al. assessed the impact of baicalein, previously studied for its anti-inflammatory, antioxidant, and anticancer potential (6), on ram semen parameters post freeze-thawing. The addition of 0.5 mM baicalein to semen extenders improved progressive motility and chromatin integrity of ram spermatozoa.

Boar semen doses are typically stored at 16–18°C, which is challenging to maintain during transport (7). Hallberg et al. comparatively evaluated boar sperm quality and fertility after storage in AndroStar Premium extender at 4°C and 16–18°C for 1 week. They found better membrane integrity in semen stored at 16–18°C, while DNA fragmentation was lower at 4°C. There was no significant difference in the number of blastocysts developed post *in vitro* fertilization between the two storage temperatures.

Dog semen cryopreservation allows preservation of gametes from individuals with significant genetic value, while also overcoming the constraints associated with traditional breeding methods (8). Domain et al. provided a comprehensive analysis of the use and popularity of frozen sperm among dog breeders in Belgium and the Netherlands, while also characterizing the individuals presented for sperm cryopreservation. The study revealed a growing trend in semen cryopreservation, with annual growth rates between 8.4 and 41.9%. Most dogs presented for cryopreservation were aged 1–9 years, and the frozen sperm was primarily intended for international shipment, although a significant portion remained unused.

Rooster semen quality declines after storage at 2–5°C for more than 24 h, likely due to oxidative stress (9). Koedkanmark et al. investigated the impact of adding *Eurycoma longifolia* (EL) extract as an antioxidant in semen extender on Thai chicken semen quality and fertility. Data indicated that supplementing the sperm cooling medium with 15 mg/ml of EL extract improved semen quality during 5°C storage for up to 48 h, by reducing lipid peroxidation, and significantly enhanced the fertility of Thai rooster semen stored for up to 24 h.

Endocrinological challenges were also approached by several authors, and their research explored various hormonal implications in the reproductive processes.

The anti-Müllerian hormone (AMH) is recognized as a valuable marker for evaluating testicular function (10). Posastiuc et al. found that higher serum levels and tissue expression of AMH are linked to smaller seminiferous tubules and poorer Johnsen scores, suggesting AMH as a marker of testicular degeneration in dogs.

Scopolamine has emerged as a viable alternative to traditional ecbolic substances in managing the postpartum period in dairy cows (11). Carbonari et al. compared the effect of oxytocin, prostaglandin F2α (PGF2α) and scopolamine on uterine involution and resumption of ovarian activity in dairy cows and concluded that treatment with scopolamine and PGF2α resulted in faster uterine involution and ovarian recovery.

Gonadotropin-releasing hormone (GnRH) is commonly used in fixed time artificial insemination protocols for sheep, although its effect on pregnancy rates continues to be a subject of debate (12). Zhang et al. evaluated its influence on pregnancy rates and pre-implantation metabolites in Huyang ewes, synchronized using a progestogen-eCG protocol. Evidence showed a significant decrease in hydroxyproline and an increase in corticosterone and prostaglandin D2 levels, correlating with lower pregnancy rates.

GnRH administration significantly decreases the pregnancy rate of recipient ewes after embryo transfer, possibly because it affects endometrial epithelial cell function (13). Jiao, Chu, et al. investigated the effect of GnRH on endometrial epithelial cells by screening the S100A4 gene transcription. Results revealed that GnRH suppresses S100A4 expression in the endometrium, consequently inhibiting endometrial cell proliferation via the S100A4/GNAI2/MAPK signaling pathway, potentially explaining decreased embryo implantation rate. Another study by Jiao, Jiao, et al. highlighted the role of GnRH in endometrial cell senescence, identifying interactions between S100A4 and PPP1CA, and its involvement in cellular senescence regulation through the S100A4/PPP1CA/IL-17 pathway.

Peripartal and postpartum management in dairy cows was also approached, thus Rafa et al. characterized the metabolic and hormonal profiles of Romanian Spotted cows during the peripartum period, exploring possible correlations with retained fetal membranes (RFM), a pathology frequently associated with economic loss, due to decreased milk production and high costs of medical treatment (14). Their findings confirmed that significant metabolic and physiological changes due to RFM occur postpartum, emphasizing the need for targeted management strategies.

Chinese herbal medicine (CHM) has shown beneficial effects on cow health and production (15). Abulaiti et al. evaluated the effects of a CHM preparation on the growth, milk yield, and reproductive efficiency of dairy cows in early postpartum period. The study found no adverse effects on biochemical indicators for immunity, digestibility, and metabolism, while improving estrus, ovulation, and pregnancy rates.

A novel method using micro-computed tomography to explore swine oviduct anatomy was developed by Belda-Perez et al. This approach provided detailed parameters like shape factor, fractal dimension, and lacunarity, enabling more precise embryo production and epigenetic reprogramming.

Finally, Nechifor et al. published a brief report on the uterine bacteriological load in Holstein Friesian cows, based on parturition type (eutocia/dystocia). They found a significant increase in germ count during the first 14 days postpartum, with higher levels in cows experiencing dystocic calving.

In conclusion, these studies collectively provide a comprehensive resource for researchers, scholars, and practitioners in veterinary reproduction and reproductive biotechnologies.
